# The influence of hydrodynamic exposure on carbon storage and nutrient retention in eelgrass (*Zostera marina* L.) meadows on the Swedish Skagerrak coast

**DOI:** 10.1038/s41598-020-70403-5

**Published:** 2020-08-12

**Authors:** Martin Dahl, Maria E. Asplund, Mats Björk, Diana Deyanova, Eduardo Infantes, Martin Isaeus, Antonia Nyström Sandman, Martin Gullström

**Affiliations:** 1 Seagrass Ecology & Physiology Research Group, Department of Ecology, Environment and Plant Sciences, Stockholm University, Stockholm, Sweden; 2 Department of Marine Sciences, University of Gothenburg, Kristineberg, Fiskebäckskil, Gothenburg, Sweden; 3 Department of Biological and Environmental Sciences, University of Gothenburg, Kristineberg, Fiskebäckskil, Gothenburg, Sweden; 4 Norwegian Institute for Water Research (NIVA), Oslo, Norway; 5 AquaBiota Water Research, Stockholm, Sweden; 6 School of Natural Sciences, Technology and Environmental Studies, Södertörn University, Huddinge, Stockholm Sweden

**Keywords:** Ecosystem services, Ecosystem ecology

## Abstract

Cold-temperate seagrass (*Zostera marina*) meadows provide several important ecosystem services, including trapping and storage of sedimentary organic carbon and nutrients. However, seagrass meadows are rapidly decreasing worldwide and there is a pressing need for protective management of the meadows and the organic matter sinks they create. Their carbon and nutrient storage potential must be properly evaluated, both at present situation and under future climate change impacts. In this study, we assessed the effect of wave exposure on sedimentary carbon and nitrogen accumulation using existing data from 53 *Z. marina* meadows at the Swedish west coast. We found that meadows with higher hydrodynamic exposure had larger absolute organic carbon and nitrogen stocks (at 0–25 cm depth). This can be explained by a hydrodynamically induced sediment compaction in more exposed sites, resulting in increased sediment density and higher accumulation (per unit volume) of sedimentary organic carbon and nitrogen. With higher sediment density, the erosion threshold is assumed to increase, and as climate change-induced storms are predicted to be more common, we suggest that wave exposed meadows can be more resilient toward storms and might therefore be even more important as carbon- and nutrient sinks in the future.

## Introduction

Carbon sequestration and nutrient retention are highly important ecosystem services provided by seagrass ecosystems^1–4^. The natural carbon and nutrient sinks these meadows provide are suggested to contribute to the mitigation of the acute threats to human wellbeing posed by climate change and increased anthropogenic pressure on the coastal zone^5,6^. Therefore, there is an urgent need to increase protection of environments with high capacity for long-term carbon storage and nutrient filtering^7,8^, where environmental managers also need to consider future effects of climate change. Seagrass meadows have been highlighted as efficient carbon and nutrient sinks^9–12^, but this ecosystem function is not constant and varies significantly among regions and species^13–15^. The ability of seagrass meadows to sequester and store organic matter is linked to their high primary production, the slow decomposition in marine sediment and their efficient trapping of allochthonous particles from surrounding habitats^16,17^. The contribution of allochthonously derived organic matter to the accumulation of sedimentary organic matter can be substantial^17,18^, as seagrasses trap organic particles by reducing the flow of the water within the canopy^19–21^ causing suspended organic matter to settle within the meadow and increasing the sedimentation rate^22^. Hydrodynamics will thus largely determine sedimentation processes in seagrass-dominated environments^23^ and it has been shown that low exposure to wind and wave action tends to favour a build-up of carbon-rich sediments^14,15,24–26^. Contemporary hydrodynamic conditions also affect the structure and morphology of the seagrass plants^27,28^. Strongly linked to the impact of wind and wave action is the water depth of the meadows, where a deeper location is likely to experience less influence from waves because of the attenuation of the hydrodynamic energy with depth^29^.

Eelgrass, *Zostera marina* (L.), is a foundation species in the cold-temperate coastal environment and is widely distributed in the northern hemisphere. Due to its large distribution range and difference in environmental conditions, *Z. marina* meadows show a large variability in carbon-^14,15,30,31^ and nitrogen storage^32^, with exceptionally high carbon stocks at the Swedish west coast^14,15^. This variability in storage capacity has been linked to sediment properties, with meadows having a high proportion of fine grain-sized particles and degree of sorting (i.e. the uniformity of sediment grain sizes), and low sediment density being related to higher carbon and/or nutrient stocks^14,15,31,33,34^. These sediment characteristics are in turn associated with areas of low hydrodynamic forces and the grain size distribution and sorting has been used as a proxy for hydrodynamic exposure^14,15,31,34^, but to what extent wave exposure governs *Z. marina* carbon and nitrogen stocks on a regional scale is not fully known. On the Swedish west coast, *Z. marina* meadows are found in a range of hydrodynamic exposure levels and water depths, from sheltered bays to areas with high exposure levels^35^ and with a depth distribution of about 1–9 m^36^. The dominant hydrodynamic forcing in the area is from waves and currents as the tidal amplitude is very low (with a daily tidal variation of approximately 0.2 m)^37^.

As seagrass areas are declining worldwide^9,38^, with the Swedish west coast having experienced a 60% decrease in *Z. marina* areal cover since the 1980s^39,40^, assessments of carbon and nutrient stocks and understanding the factors influencing the storage function are needed in management and protection of seagrass organic matter sinks for prioritization of conservation efforts (as well as for other ecosystem services provided by seagrasses^41^). Management of *Z. marina* also needs to consider future effects of climate change, as storms are likely to increase in intensity and frequency^42^, which could cause erosion and negatively impact the sink function (based on the storage of carbon and nitrogen), especially in meadows with muddy low density sediment^43^. In this study, we used a depth-attenuated wave exposure model (Simplified Wave Model, SWMd), which models the long-term hydrodynamic exposure level along the seabed^29^. Wave direction and energy is usually highly variable over shorter time periods, while displaying higher stability when analyzed over a longer temporal scale. Wave exposure is thus difficult to measure on site, and is therefore usually calculated using different indices. The SWMd has been proven to have high ecological relevance compared to other wave models^44^. The overall aim was to assess to what extent wave exposure (based on the SWMd) affects (1) sedimentary carbon and nitrogen content (as percent dry weight [DW]), and (2) stocks (g m^−2^) in *Z. marina* meadows on the Swedish Skagerrak coast. Based on the findings of previous studies on the effect of hydrodynamics on seagrass sedimentary carbon, where they found higher carbon- and/or nitrogen storage in more sheltered areas^24,25,43^, we hypothesised that meadows with lower hydrodynamic exposure will have higher sedimentary carbon and nitrogen content (in percent DW) and stocks (g m^−2^). To test this hypothesis, we first assessed the relationship between wave exposure and the percent (of DW) carbon and nitrogen content in surface sediment (0–5 cm) in 53 eelgrass meadows along the coast of Bohuslän in Sweden, and secondly, explored the effects of hydrodynamic conditions on carbon and nitrogen stocks (g m^−2^, 0–25 cm) on a smaller subset of seven meadows. We also assessed the relationship between grain size properties (i.e. mud content and degree of sorting) and hydrodynamics as these sediment characteristics have previously been linked to high sedimentary organic carbon content in *Z. marina* meadows^14,15,31,34^.

## Results

### Relationships between carbon- and nitrogen content in surface sediment (0–5 cm) and wave exposure

**Figure 1:**
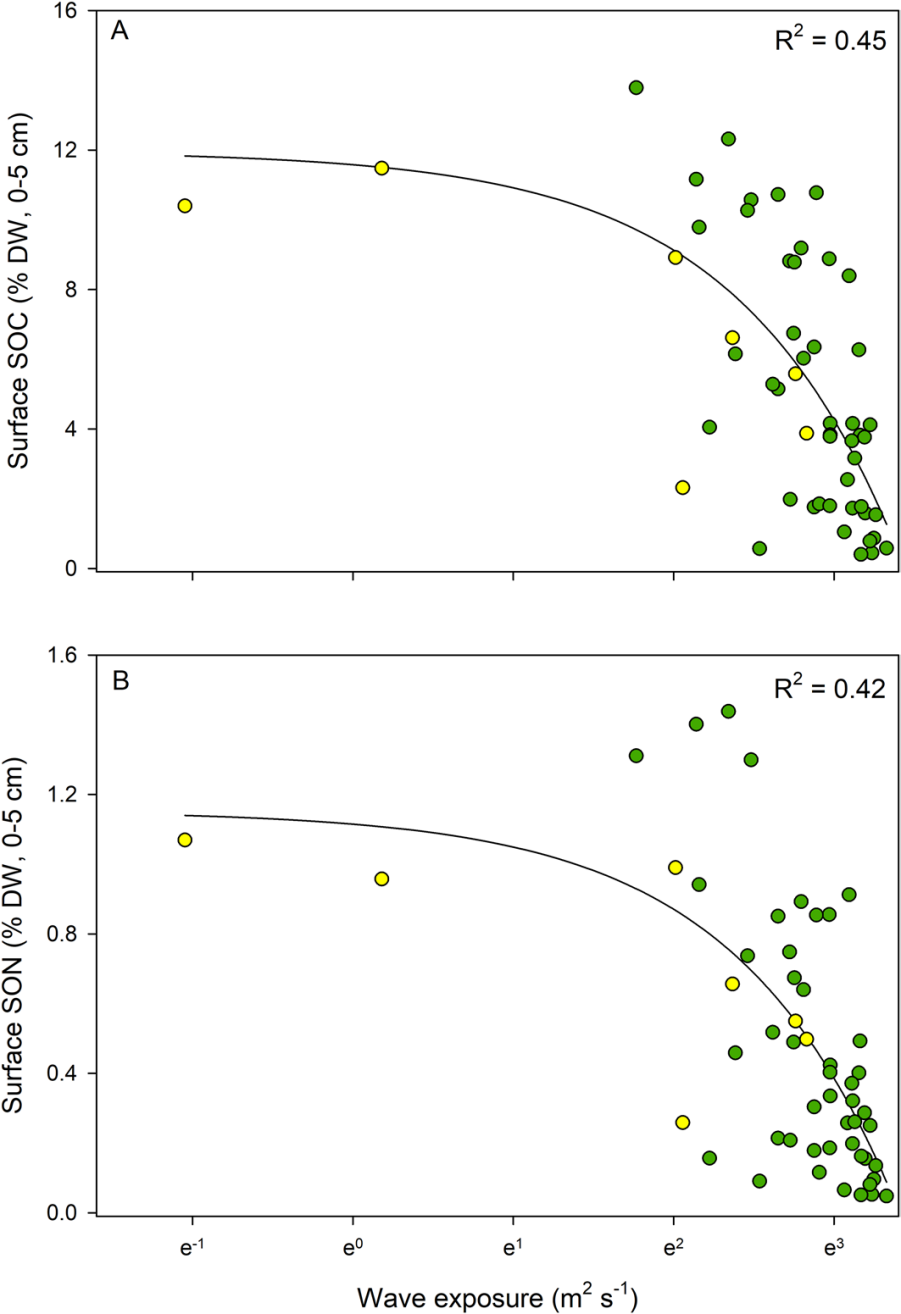
Relationships between wave exposure and percent dry weight (DW) of sedimentary organic carbon (SOC) (**A**) and nitrogen (SON) (**B**) (0–5 cm) for all sites (n = 53). The yellow dots (n = 7) represent the sites (the deeper part of the meadow) where the longer cores (0–60 cm) were collected. The values of the SWMd are expressed in a natural logarithm scale and non-logarithmic values ranged from 3 × 10^–9^ to 2,552 m^2^ s^−1^ in the most exposed site.

When correlating the percent (% DW) carbon and nitrogen of the surface sediment (0–5 cm) with the hydrodynamic exposure for all sites (n = 53), we found that there were inverse relationships between wave exposure and both surface sedimentary organic carbon (SOC) (d.f. = 51, F = 43.11, p < 0.001) and nitrogen content (SON) (d.f. = 51, F = 38.43, p < 0.001) (R^2^ = 0.42–0.45) (Fig. 1). The two sites with the lowest wave exposure gave the impression to be the main drivers of the trends seen in Fig. 1a,b, but when this was tested by removing these two sites, the significance level did not change and there was only a slight decrease in the variation to the fitted regression (with a R^2^ of 0.4 for SOC [% DW] and 0.38 for SON [% DW]; Fig. S1a,b). Seagrass morphology and the associated function of water attenuation can be affected by the hydrodynamic conditions; however, there were no correlations between aboveground biomass or shoot density and wave exposure (Fig. S2a,b).

### Influence of wave exposure on carbon- and nitrogen stocks (0–25 cm)

**Figure 2:**
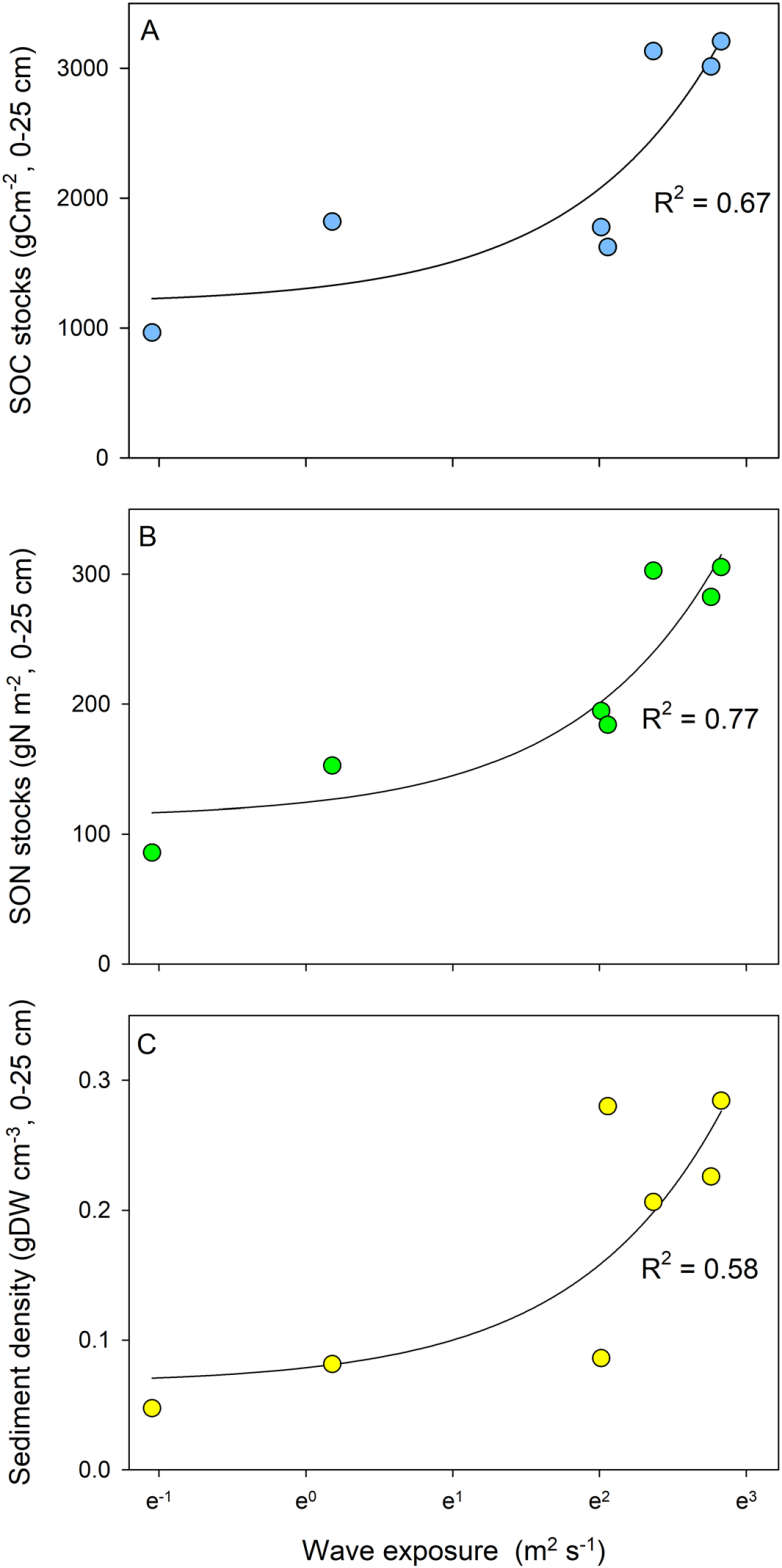
Wave exposure in relation to (**A**) sedimentary organic carbon (SOC) and (**B**) nitrogen (SON) stocks (g m^−2^, 0–25 cm), and (**C**) sediment density (gDW cm^−3^, 0–25 cm) for the deeper part of the meadows (n = 7). The carbon stocks were corrected for sediment compression during sampling. The values of the SWMd are expressed in a natural logarithm scale and non-logarithmic values are presented in Table 1.

Assessments of carbon and nitrogen stocks in relation to the hydrodynamic exposure for the sites with longer cores (0–25 cm) showed that sedimentary carbon- (d.f. = 5, F = 13.0, p < 0.05) and nitrogen stocks (g m^−2^) (d.f. = 5, F = 20.6, p < 0.01) were positively correlated to wave exposure in the deeper part of the meadows (Fig. 2a,b), which was the opposite to what was seen for the % DW carbon and nitrogen content in the surface sediment (Fig. 1). In the shallow part of the meadows, however, no relationships were seen (Fig. S3b,c). The average (± SE) carbon and nitrogen stocks were 2,219 ± 335 gC m^−2^ and 215 ± 32 gN m^−2^, respectively (for site-specific stock data, see Table 1), which are in the range of previous estimates for the Skagerrak area^15,33^. The sediment density (gDW cm^−3^) in the deeper part of the meadows was also positively correlated to wave exposure (d.f. = 5, F = 9.19, p < 0.05; Fig. 2c), while no relationship was found for the shallow parts of the meadows (Fig. S3c).

**Table 1:** Summary of sedimentary organic carbon (SOC) and nitrogen (SON) stocks, water depth and modelled wave exposure in the deeper part of the studied *Z. marina* meadows.

Site	SOC stocks (g C m^−2^, 0–25 cm)	SON stocks (g N m^−2^, 0–25 cm)	Water depth (m)	SWMd (cm^2^ s^−1^)
Getevik	947	45	4	0.00003
Sladholmen	1979	150	3	0.00007
Lindholmen	1815	191	2.7	0.03639
Kristineberg	1534	158	4	0.05149
Rixö	3,159	297	3	0.88351
Styrsvik	3,019	279	3	150.20589
Trommekilen	3,334	301	2.5	464.34990

*SWMd* depth-attenuated Simplified Wave Model.

### Sediment grain size properties in relation to wave exposure

**Figure 3:**
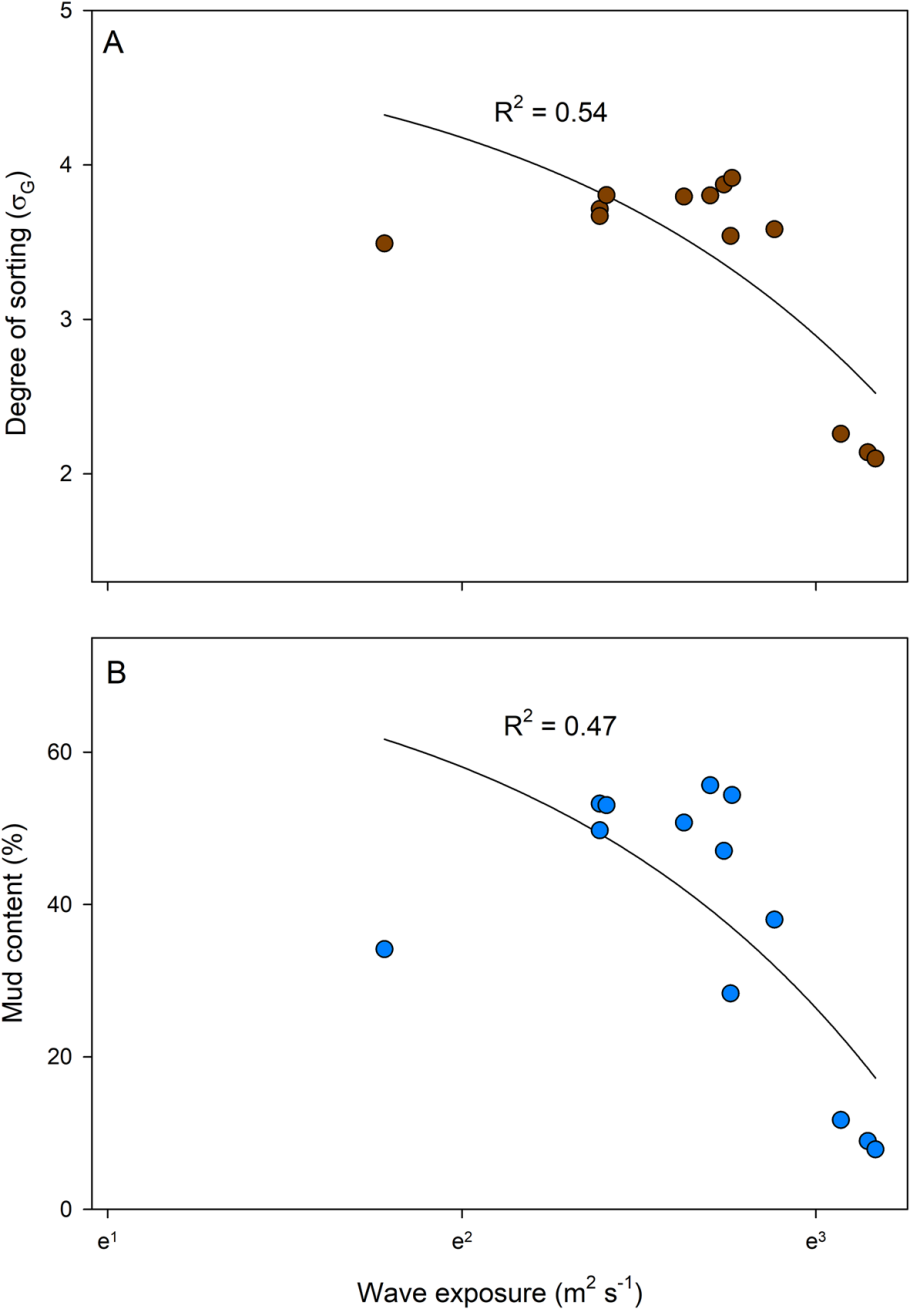
Relationships between wave exposure and (**A**) degree of sorting (σ_G_), and (**B**) mud content (% DW) in surface (0–1 cm) sediments (n = 13). The values of the SWMd are expressed in a natural logarithm scale and non-logarithmic values ranged from 0.0001 to 43 m^2^ s^−1^ in the most exposed site.

**Figure 4:**
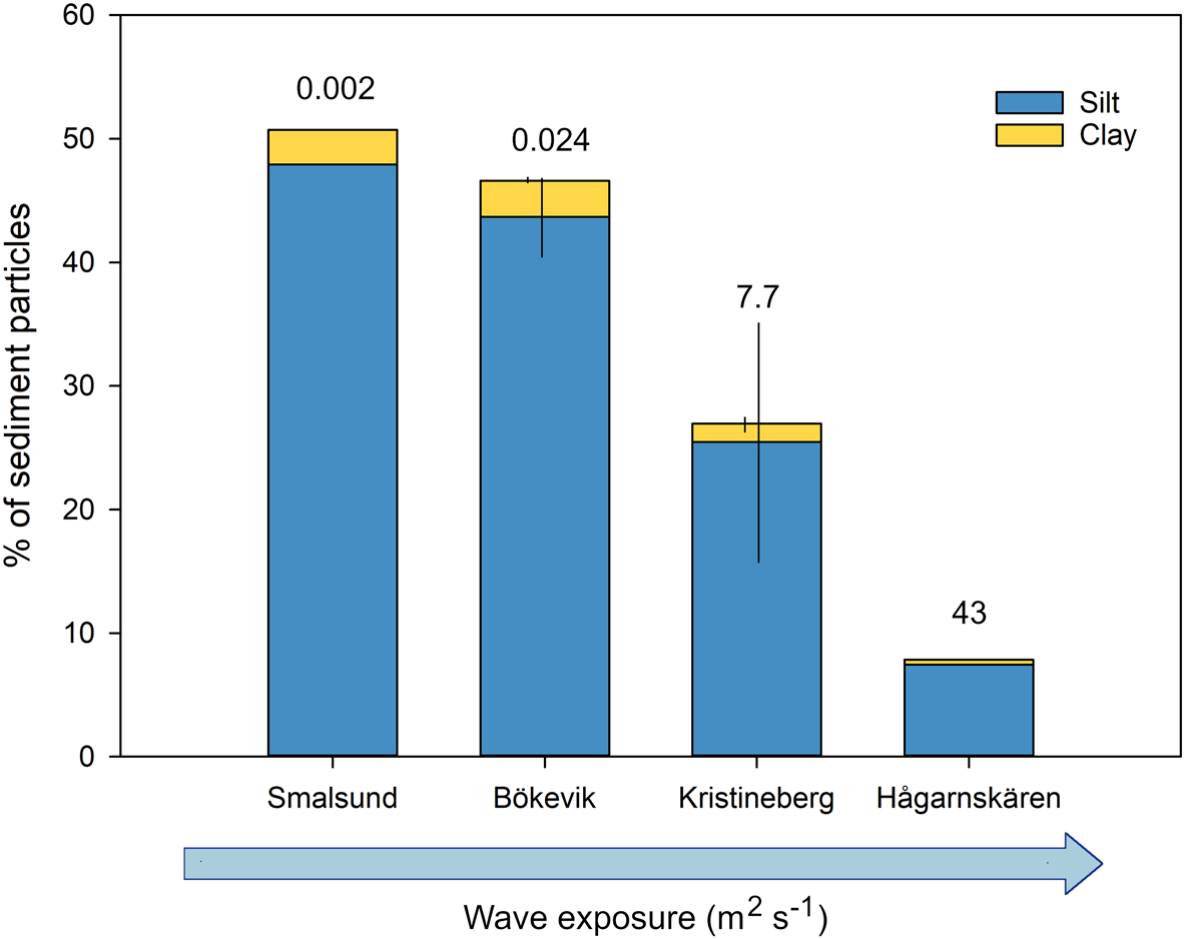
Mean (± SE) percent dry weight mud content separated into silt (4–63 µm) and clay (< 4 µm) fractions in the top 1 cm of sediment in *Z. marina *meadows at four sites with different hydrodynamic exposure. The values above the bars indicate the wave exposure level of the sites (m^2^ s^−1^).

Correlative analyses between grain size properties for the surface sediment (0–1 cm) and wave exposure (based on 13 sites with different hydrodynamic exposure levels) showed that both mud content (% DW) and degree of sorting (σ_G_) of the surface sediment were negatively correlated to wave exposure (d.f. = 11, F = 11.5, p < 0.01 and d.f. = 11, F = 15.1, p < 0.01, respectively, Fig. 3). The mud content (i.e. silt and clay fractions combined) was higher in the more sheltered sites and the main fraction of the mud content was silt with only a minor proportion of clay particles in any of the sites (ranging from 0.4 to 2.9%) (Fig. 4).

### Influence of sediment depth on carbon and nitrogen content

**Figure 5:**
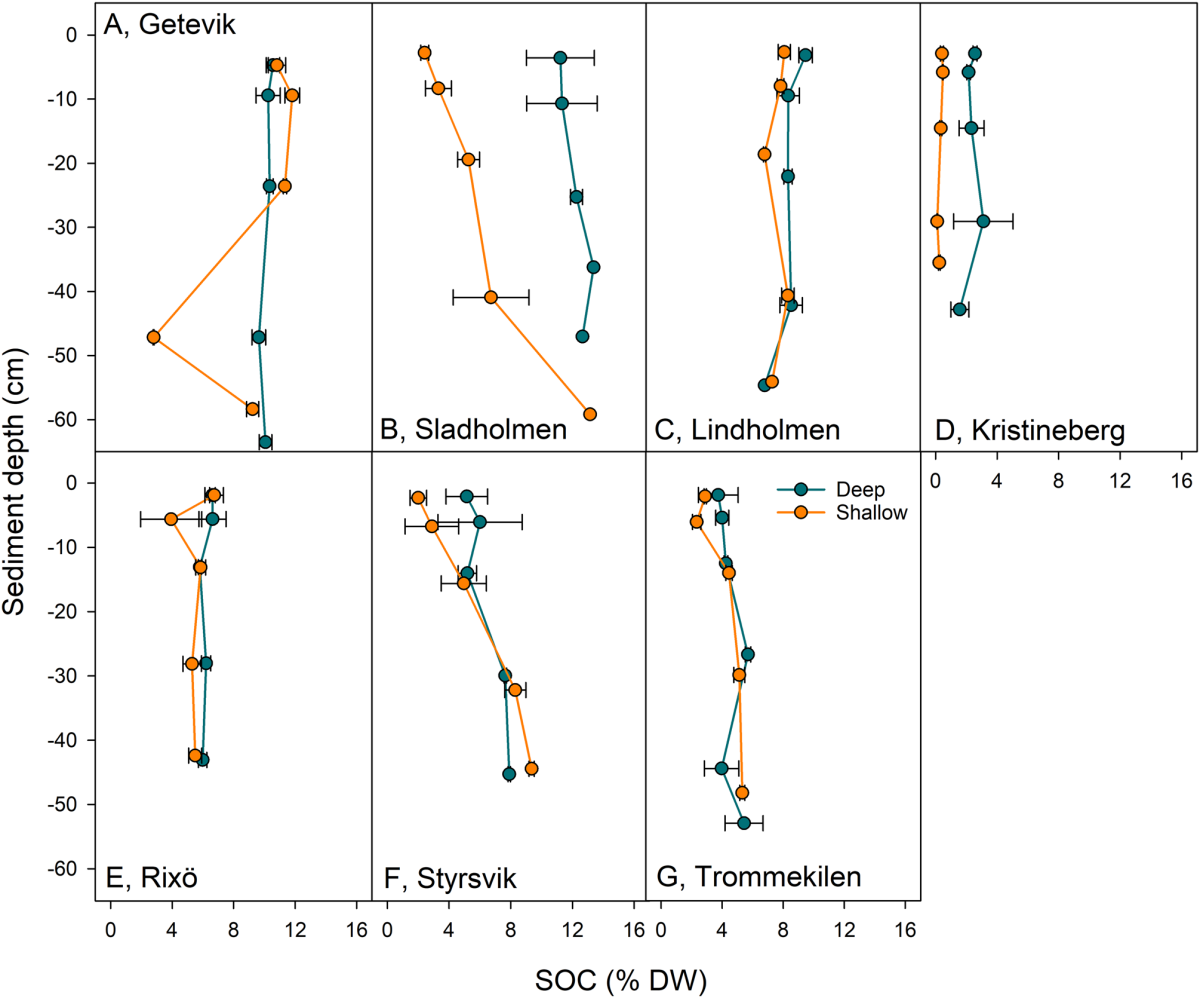
Mean (± SE) sediment depth profiles of sedimentary organic carbon (SOC) (% DW) in the deep and shallow parts of the meadows. The sediment depths were corrected for sediment compression during sampling.

In the sites with longer cores (n = 7), there were no changes along the sediment profiles in either shallow or deeper parts of the meadows for SOC (Fig. 5) or SON content (% DW), nor for sediment density (g m^−2^) or C:N-ratio (carbon:nitrogen density-ratio) (Fig. S4).

## Discussion

The main findings from this study show that hydrodynamic exposure significantly affects the organic matter sink function in *Z. marina* ecosystems and that low wave exposure does not necessarily translate into higher organic matter storage. The mechanism behind this is that in hydrodynamically exposed meadows the packing density of the sediment becomes higher, causing the specific weight of the sediment to increase, which in turn concentrate the carbon and nitrogen stocks. Previous studies have shown an opposite pattern^24–26^, however, our results, using core compression-corrected sediment densities down to 25 cm and the low-density sediment bed types found in this region of the Swedish west coast, show that when relating wave exposure to the levels of carbon and nitrogen stocks we find a reversed relationship. This is explained by a reduced sediment density (and associated carbon- and nitrogen density) in the most sheltered sites, and although high in carbon and nitrogen percent content per dry weight, this results in a reduction of the organic matter stocks per volume, and highlights the need of including the sediment density when evaluating organic matter storage. These findings are of importance for management of cold-temperate seagrass carbon storage and could be used to assess the resilience towards climate change impacts, as the meadows with higher sediment densities and associated carbon and nitrogen stocks will likely better withstand the projected increase in storms.

Sediment characteristics are considered the most important factors for carbon storage in *Z. marina* meadows, whereas seagrass structural complexity and biomass properties, such as canopy height and plant dry weight, have been found to be of minor relevance^14,15,25,31,45^. This also explains why seagrass biomass has a relatively low contribution to the sedimentary organic carbon pool in *Z. marina* meadows^15,46^ and highlights the importance of hydrodynamic exposure and transport of allochthonous organic matter for the carbon sink function in these environments. In fact, seagrass structural complexity influences the hydrodynamic forces by reducing water velocity within the meadow^20^, while at the same time exposure to hydrodynamic forces can influence the meadow properties^27^ and morphology of the seagrass plants^28,47^. For instance, long and flexible canopy can cause less stress on the anchoring root- and rhizome system^48^, while larger shoots can also cause increased drag and uprooting in bed types with low sediment density^49^, which are common on the Swedish Skagerrak coast^14^. However, there was no indication that seagrass biomass or shoot density was correlated to wave exposure in this study. Hydrodynamics are to a large degree controlling the sedimentation processes^50^ and higher hydrodynamic forcing causes increased carbon (and likely nitrogen) erosion on the sediment surface^43^; therefore, it was not surprising that the sediment carbon and nitrogen per sediment dry weight was negatively associated with wave exposure, as indicated previously^14,51^. Similarly, sediment with low density and high mud content, which was found in hydrodynamically sheltered meadows, has previously been linked to higher carbon storage in both temperate and tropical seagrass meadows^14,15,31,45,52^, as organic matter generally has lower weight than minerogenic material^50^, and contains more water^53^. However, a higher water content reduces the compaction and negatively affects the stability of the sediment^50,54^ and consequently, a low sediment density is lowering the erosion threshold^55,56^. The sediment grain size properties in several of the meadows showed that the high mud content (i.e. the silt and clay fractions) was mainly composed of silt particles and a low proportion of clay (< 3%), which is below the threshold level (5–10%) considered for cohesive sediment bed types^57^. Hence, the sediment of these meadows is likely easily erodible as the cohesiveness is of importance for stabilizing the sediment^54^. In this intrinsically unstable sediment bed type, the seagrass plants’ ability to protect the sediment from resuspension and erosion also becomes impaired^43,58^. Coarse sediment with higher density and compaction is found in more hydrodynamically exposed environments^59,60^, which explains why the sediment density increases relative to the wave exposure, as seen in this study. The increase in the density and weight of the sediment subsequently raises the carbon and nitrogen stocks, despite the inverse relationship of the percent carbon and nitrogen content per weight with hydrodynamic exposure. Both carbon and nitrogen (as percent per dry weight and stocks per volume) have been used in the literature (e.g.^10,13,15^) to estimate the storage capacity of seagrass meadows. However, we found an opposite pattern to the hydrodynamic exposure level of these two carbon and nitrogen measurements, and by considering also the density of the sediment a better representation of the organic matter storage can be provided.

**Figure 6:**
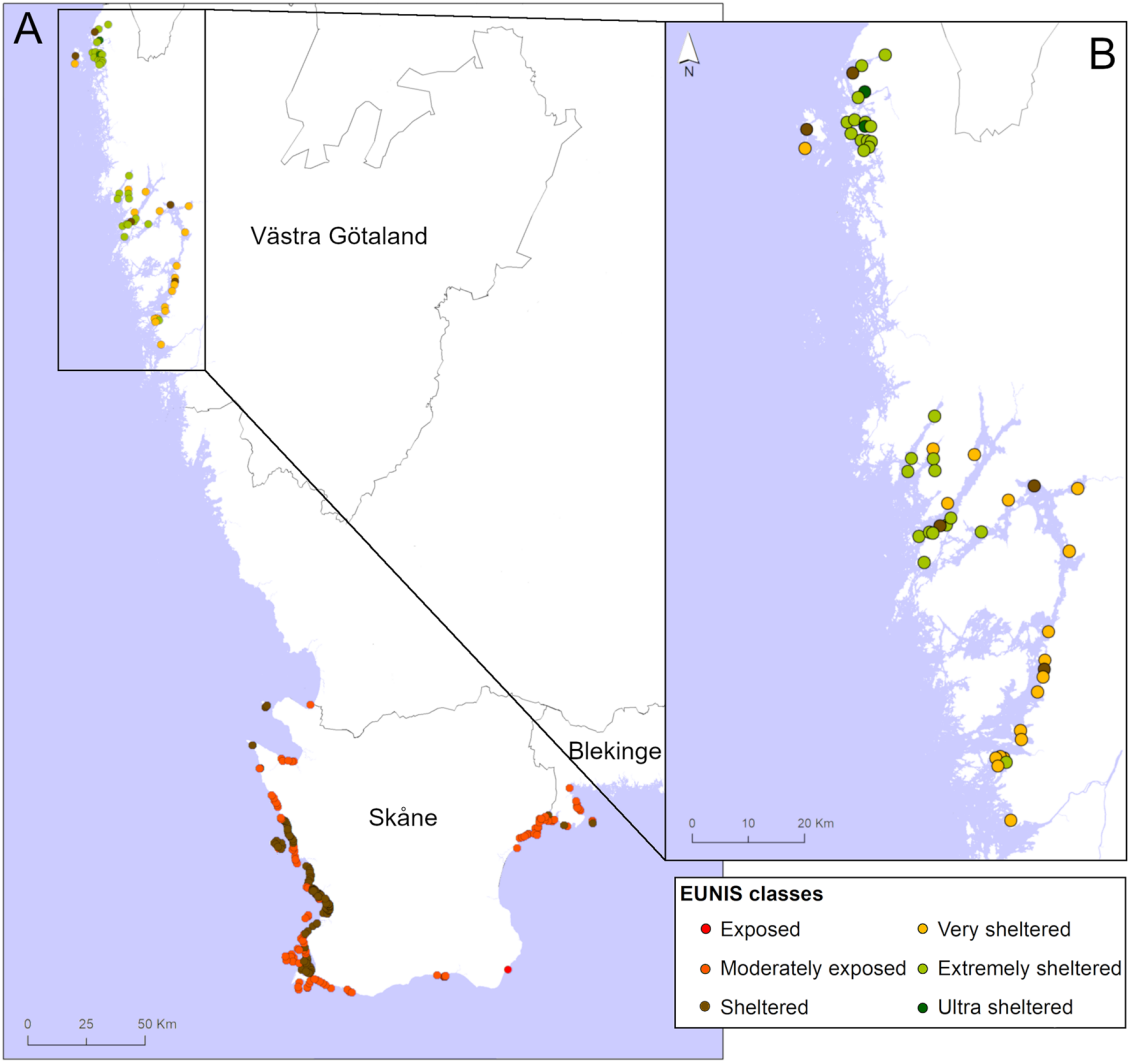
Wave exposure levels based on the EUNIS classification^61^ for seagrass sites (**A**) along the Swedish west coast in general and (**B**) the Swedish Skagerrak coast, where the sampling of this study was conducted. The classification is based on the surface wave exposure and is not corrected for water depth. The seagrass sites in Skåne and Blekinge were retrieved from the citizen science programme SeagrassSpotter (https://seagrassspotter.org/). The map was generated using the ArcGIS software (v. 10.7) (https://www.esri.com).

When comparing the exposure levels, based on the EUNIS (European Nature Information System) classification scheme^61^, for the *Z. marina* meadows in this study to other seagrass areas along the Swedish west coast (Fig. 6) and the Baltic Sea^62^, it is clear that the meadows in this study are within the lower range of hydrodynamic exposure, with the most frequent exposure level being “extremely sheltered” (n = 28 of the studied sites; Fig. 6b). There is likely an upper threshold for the wave exposure, where the hydrodynamic forces become strong enough to cause significant erosion and export of organic particles^43^ and thereby reducing the organic matter storage. This has been suggested as an explanation for the low carbon stocks found in the Baltic Sea^31^, and is also likely the reason for the negative association of wave exposure to carbon stocks found in other studies^24–26^. However, the previous studies are based on different methods for examining the relationship between hydrodynamic exposure and carbon stock levels, making direct comparisons difficult. They either used a model approach^24,26^, which is acting on a landscape scale (10 s to 100 s km) and long temporal scales (months to years), or used plaster of Paris chalk blocks^25^, which measure integrated hydrodynamic conditions on a meadow (km) scale and over a shorter time period (days to weeks). The models either estimated the depth-average current velocity^24^ or wave height^26^ and assess two different aspects of hydrodynamic forces (i.e. unidirectional and oscillatory flow). The model approaches used in these previous studies as well as in this study are reflecting the hydrodynamic conditions on large spatial and temporal scales, which are likely the scales most important for carbon and nitrogen storage as well as for management of organic matter sinks.

Only the deeper parts of the studied *Z. marina* meadows (2.5–4 m depth) displayed an influence of hydrodynamic exposure, likely due to the narrow range of wave exposure levels in the shallow parts, where the water depth was highly similar (varying between 1.3 and 1.5 m). The shallow parts of the meadows were also in the higher exposure range as the wave exposure rapidly decreases with depth in the model, although showing comparable carbon and nitrogen stocks as in the deeper parts. This indicates that other factors, such as seagrass productivity^63^, are influencing the carbon- and nitrogen storage in the shallow parts of the meadows. The influence of turbidity on the seagrass productivity might be of importance, considering the susceptibility for resuspension in the low density and non-cohesive sediment found in many of the meadows, and therefore the light irradiance might not only be affected by the attenuation of the water but also the amount of sediment particles. There were no changes in carbon and nitrogen content along the sediment depth profiles down to the maximum depth of about 60 cm, which strengthens the argument that the surface content (0–5 cm) sampled for the 53 meadows in the large survey is valid for evaluating meadows for long-term carbon storage, as similar values between the surface- and deeper sediment layers are expected. However, this might also indicate that there is a mixing of the sediment layers^33^, for instance from bioturbation, as the organic matter content would otherwise decrease due to remineralization^64^. Therefore, the upper 50–60 cm of the sediment might potentially still be within the remineralization zone^65^, with declining carbon and nitrogen content and refractory accumulation occurring in deeper layers of the sediment^51^. This also highlights the need for sampling of longer cores deeper than 60 cm to properly assess the long-term accumulation and storage of carbon and nitrogen in these environments. Sediment density is not only related to organic matter content^53^, but increases with time and sediment depth^66^, which indicates that accumulation rate of organic matter might be higher in the sheltered sites. Hence, these meadows could show a higher organic matter sink capacity despite having a low sediment density when sampling of the total carbon and nitrogen accumulated in a meadow and accounting for the entire sediment thickness. In this study, we used a standardized sediment depth of 25 cm as the actual sediment deposit for the meadow was not available, and therefore, we also highlight the need for measuring the total sediment depth when evaluating the carbon and nitrogen sink function of seagrass meadows.

Storms, however, might alter the relationship between sediment accumulation and erosion. The amount of storms have likely increased in temperate regions over the last century^67^ and the intensity and frequency are projected to increase even more in the future due to the ongoing climate change^42^. An increase in storm intensity and frequency can severely impact the health of the seagrass ecosystem and negatively affect the organic matter sink function, as storm events may lead to mechanical damage to the meadows^68,69^, burial of the seagrass plants^70^, increased resuspension^71,72^ and turbidity^73^, and erosion of sedimentary organic carbon^43^. Storms could, therefore, as a result of the erosion of the sediment, increase the CO_2_ emission as ancient carbon is being remineralized and enhance the nutrient load of the local coastal environment. Many of the meadows displayed an unstable sediment bed type with low compaction and clay content, and low-density *Z. marina* sediment are more sensitive to abrupt increases in flow velocity (such as during a storm event) and display a higher organic carbon resuspension compared to more densely compacted sediment^43^. Therefore, meadows with high sediment density are likely to be more resilient towards storms with less of the carbon- and nitrogen stocks being eroded.

This large-scale assessment with multiple meadows highlights that management of *Z. marina* meadows as organic matter sinks should focus on landscape scale processes governing transport of organic matter and sedimentation, as these are likely the main drivers for carbon- and nitrogen storage, which previous studies have also highlighted^15,23,52^. Today, there is no nationwide monitoring program for *Z. marina* in Sweden, even though the habitat has decreased considerably the last decades^39,40^ and is listed as “unfavourable—bad” in the EUs Habitat Directive. The large losses of *Z. marina* meadows since the 1980s have likely come with a major socioeconomic cost^51^ as the economic value of seagrass carbon storage and nutrient retention is substantial^2,74^. In the context of the Swedish west coast, nutrient cycling has been estimated to a value of about $ 20,000 USD/ton stored in seagrass meadows and to approximately $ 120 USD/ton for carbon^51^. In order to develop reliable predictor models for management and monitoring, a high proportion of the variation (more than 80%) needs to be explained^75,76^, and although wave exposure had a 67–77% explanation power for the carbon and nitrogen stock variations, hydrodynamics could be used together with other indicators, such as anthropogenic activity in the surrounding area and sediment characteristics, to evaluate *Z. marina* meadows of high interest as organic matter sinks. In addition, even though this study assessed the carbon- and nitrogen stocks down to a standardized depth of 25 cm, we suggest that the total sediment depth of the meadows should be evaluated in relation to exposure levels as both sediment depth and sedimentation rate can still be higher in more sheltered sites, possibly resulting in a higher total carbon- and nitrogen storage. Hydrodynamic exposure is also influencing the sediment grain size properties and in more sheltered sites the sorting of sediment particles and mud content are higher. The degree of sorting of sediment has previously been used as an indirect measure for level of hydrodynamic forces on *Z. marina* meadows^15,31^ and in our study this factor showed a moderate relationship (54%) to wave exposure. A similar relationship was seen for mud content (47%) and we suggest that these sediment characteristics could be of use in management as a proxy for hydrodynamic exposure, if no direct measurements of hydrodynamics are available. For management of *Z. marina* meadows, future climate change impacts, such as storms, need to be considered. This study shows that meadows of higher hydrodynamic exposure level had a larger carbon- and nitrogen storage, due to the more compacted sediment layers (down to 25 cm). These meadows are also likely more resilient towards storms as higher sediment densities are also less prone to erosion^43^ and might therefore play an increasingly important role for carbon and nutrient storage in the future.

## Methods

### Study area

**Figure 7:**
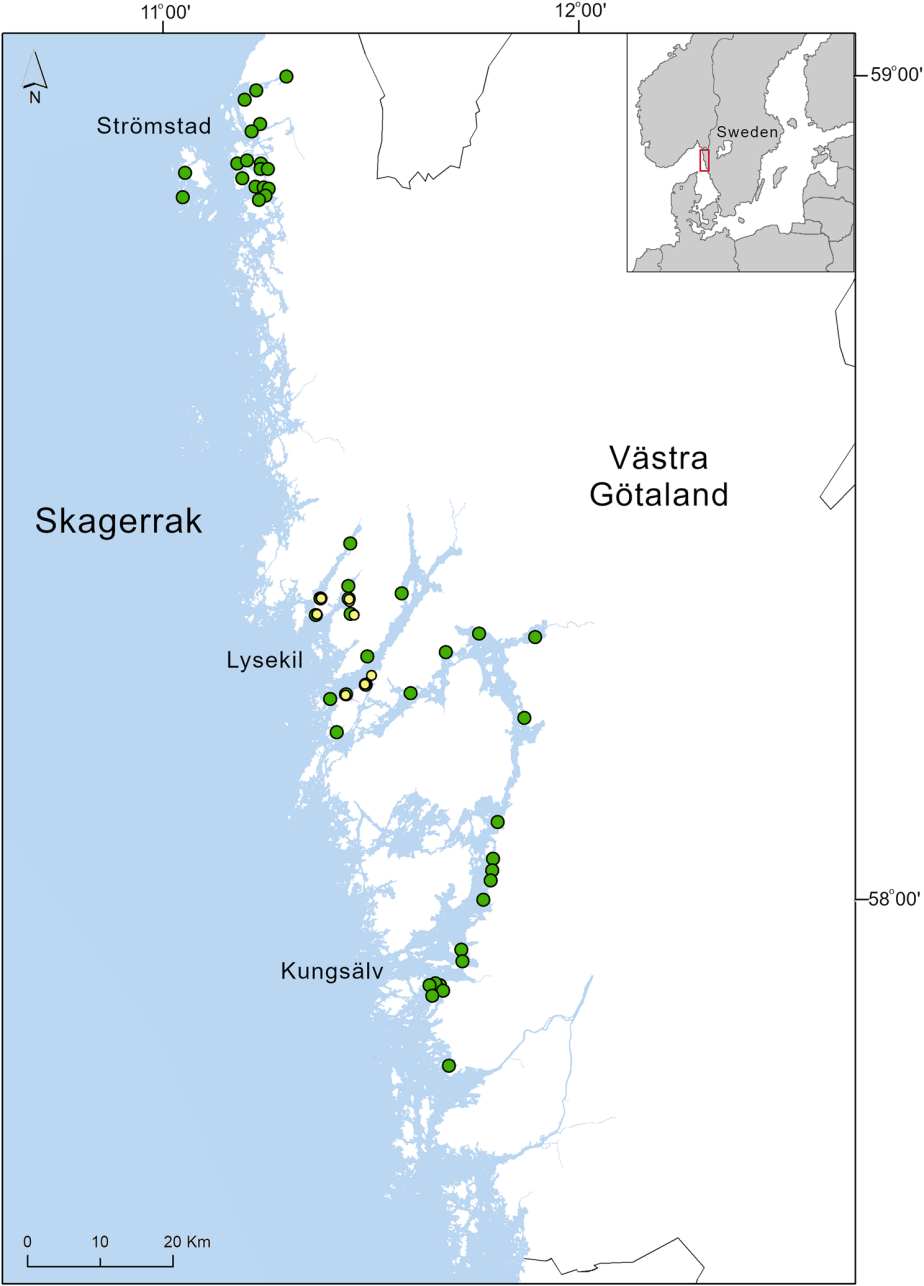
Map of the Swedish Skagerrak coast showing the sampling sites. The green dots (n = 53) present sites of *Z. marina* meadows where surface sediment (0–5 cm) was sampled, while the yellow dots (n = 7, selected among the 53 sampling sites) show locations of sites (the deeper part of the meadows) where long sediment cores (0–60 cm) were collected. The map was generated using the ArcGIS software (v. 10.7) (https://www.esri.com).

In total, 53 eelgrass (*Zostera marina*) meadows along the Skagerrak coast of Sweden were used in this study (Fig. 7), with sediment sampled during several occasions; the main sampling was conducted from late July through August 2000 (n = 42)^77^, with additional sampling conducted in June 2013 (n = 1)^14^, August 2015 (n = 2)^65^, June 2016 (n = 3)^43^ and August 2018 (n = 5). During the field sampling in 2000, 2003, 2013 and 2016 only short cores (0–5 cm) were used, while in 2015 and 2018 long sediment cores were collected (0–60 cm) and at two different water depths within each meadow, defined as shallow (1.3–1.5 m) and deep (2.5–4 m), while the water depth for the short cores (0–5 cm) ranged from 1.2 to 4.5 m. The sites sampled for long cores in 2015 and 2018 were selected to be representative for the hydrodynamic exposure levels in seagrass meadows on the Skagerrak coast, ranging from sheltered to exposed areas.

### Field sampling and analysis of carbon and nitrogen content

All sediment sampling was carried out in a representative area of the meadow, avoiding e.g. the meadow edge^78^. The sediment sampling in 2000 was carried out using a corer of 1 cm in diameter that was pressed down to 5 cm sediment depth (n = 6 in each meadow)^77^, using a similar sampling technique as Santos et al.^24^, while in 2013–2018 the push-corer technique and SCUBA diving were used for sampling of longer sediment cores (60 cm in length and 5 cm in diameter) (n = 3–6 in each of the shallow and deep parts of the meadow). For all of the meadows, water depth was recorded at the location of the sediment sampling. The longer (0–60 cm) cores were sliced at 2.5, 5, 12.5, 25 and 37.5 cm depth intervals along the sediment profiles. For some of the cores, the last interval (37.5–60 cm) was missing due to difficulties in pressing down the core to its maximum depth. The push-corer was sharpened at the edge to reduce the friction (and core shortening) and to ease the shredding of the seagrass root-rhizome complex when pressing it down into the sediment. Core shortening was recorded for each core by measuring the outer length and comparing this with the recovered length of the core; the difference was used to calculate a correction factor for sediment compression during sampling, which was used to adjust the sediment densities of the cores^79^ (Eq. 1):

1 $$DCSD= \frac{\left(SL\times CF\right)\times SV}{SW}$$

where *DCSD* is the decompressed sediment density, *SL* is the slice length, *CF* is the calculated correction factor, *SV* is the slice volume and *SW* is the slice weight.

The average (± standard deviation) compression of the sediment was 42 ± 15%. Prior to analysis of carbon and nitrogen content, belowground seagrass biomass and larger stones and shells (if any) were removed from the sediment samples and dried at 60 °C for approximately 24 h. The sediment slices were weighed to obtain the wet weight of the entire depth interval, and a subsample was weighed before and after dried at 60 °C for c. 48 h. The difference between the wet- and dry weight for the subsample was used for calculating the dry weight of the entire sediment slice. The dried sediment was subsequently homogenized and grounded using a mixing mill (Retch 400) or with mortar and pestle. The sediment density was only available from the long (60 cm) cores and was derived from the dry weight and volume of the sediment layers (gDW cm^−3^). A dried subsample of the sediment was treated with 1 M hydrochloric acid (HCl) (by direct addition until the carbonate reaction was complete) to remove inorganic carbon following the protocol of Dahl et al.^14^. As the inorganic carbon content is generally low in this environment^65^ only a minor addition of HCl was needed. After the treatment, the sediment was dried in 60 °C for approximately 12 h and analysed for organic carbon and nitrogen content (as % DW) using a CN elemental analyser (Flash 2000, Thermo Fisher Scientific). The sediment and organic carbon and nitrogen densities (g cm^−3^) were calculated from the total dry weight of the sediment samples, including belowground biomass fragments, stones and shells, as this constitutes a part of the sediment and the sediment volume (although to a minor extent). Aboveground seagrass biomass and shoot density were assessed in each of the seagrass meadows sampled in 2000. The aboveground biomass was collected using a mesh bag (n = 3) and the seagrass shoots were cut parallel to the sediment surface^77^. The shoots were counted and the biomass dried at 60 °C for 24–48 h and subsequently weighted to obtain the dry weight.

### Grain size analysis

The mud content and degree of sorting were analysed for 13 sites (using data from Dahl et al.^43^). The degree of sorting is here defined as the homogeneity of the sediment grain sizes. The sediment was collected from the topmost cm using a cut-out syringe and dried at 60 °C for approximately 48 h. Grain size was determined with a Mastersizer 3,000 particle size analyser (Malvern Instruments) and the fractions of the grain sizes were calculated as percent mL^−1^. The mud content is given as the size fraction between 0 and 63 µm (i.e. the clay and silt fractions) and degree of sorting was calculated using the equation by Folk and Ward^80^.

### Wave exposure model calculations

Wave exposure is here defined as the long-term spatial pattern of wave climate structuring species composition and distribution in shallow areas^81^. The SWM gives a continuous spatial estimate of wave exposure at the surface based on physical parameters of fetch and wind speed, mimicking refraction and diffraction effects of waves passing islands and coastline curvatures^82^. The SWM was further developed by Bekkby et al.^29^ to include attenuation of wave energy with depth; the more advanced wave exposure model (SWMd) estimates wave exposure at the seabed. Wave exposure estimates (m^2^ s^−1^) according to SWM were calculated with the software WaveImpact 1.0^82^. The wave exposure was calculated using binary grids of land and sea at 10 m spatial resolution, based on hourly wind data from 2006 to 2016 for 37 stations (downloaded from the Swedish Meteorological and Hydrological Institute, https://www.smhi.se/data/meteorologi/vind), which were averaged over 16 compass directions, each representing an angular sector of 22.5°. For every sea area, a grid cell of the map was calculated as the mean of fetch in 16 directions multiplied with average wind speed from the same direction. The depth-attenuated wave exposure (SWMd) was then calculated for all sites according to the equations in Bekkby et al.^29^.

### Statistical analysis

Prior to statistical analysis, the response variables (carbon and nitrogen content in terms of percent and stocks) were checked for normal distribution and homogeneous variances. A natural logarithm of the SWMd values were used in the analysis to avoid difficulties caused by the exponential nature of the wave exposure scale. All statistical analyses were performed in R version 2.15.3 (R Core Team 2013). To test the relationship between wave exposure and sediment-related variables—i.e. carbon and nitrogen content and stocks, sediment density, mud content and degree of sorting—linear regressions were performed. The sediment carbon and nitrogen content were averaged for each site prior to statistical modelling. Changes in carbon and nitrogen content and density with sediment depth were tested using a linear mixed-effect model (lme4 package) with site as a random factor.

## Supplementary information

Supplementary Dataset.

Supplementary Figures.

## Data Availability

All data generated or analysed during this study are included in this published article (and its Supplementary Information files).
