# Supplementary material for: The influence of hydrodynamic exposure on carbon storage and nutrient retention in eelgrass (Zostera marina L.) meadows on the Swedish Skagerrak coast
Source: Sci Rep. 2020 Aug 12;10:13666. doi: 10.1038/s41598-020-70403-5 (PMC7423977; doi:10.1038/s41598-020-70403-5)
Supplement: Supplementary file 2 — Supplementary Figures. [file 41598_2020_70403_MOESM2_ESM.docx]

**The influence of hydrodynamic exposure on carbon storage and nutrient retention in eelgrass (*Zostera marina* L) meadows on the Swedish Skagerrak coast**

Martin Dahl, Maria E. Asplund, Mats Björk, Diana Deyanova, Eduardo Infantes, Martin Isæus, Antonia Nyström Sandman and Martin Gullström

**
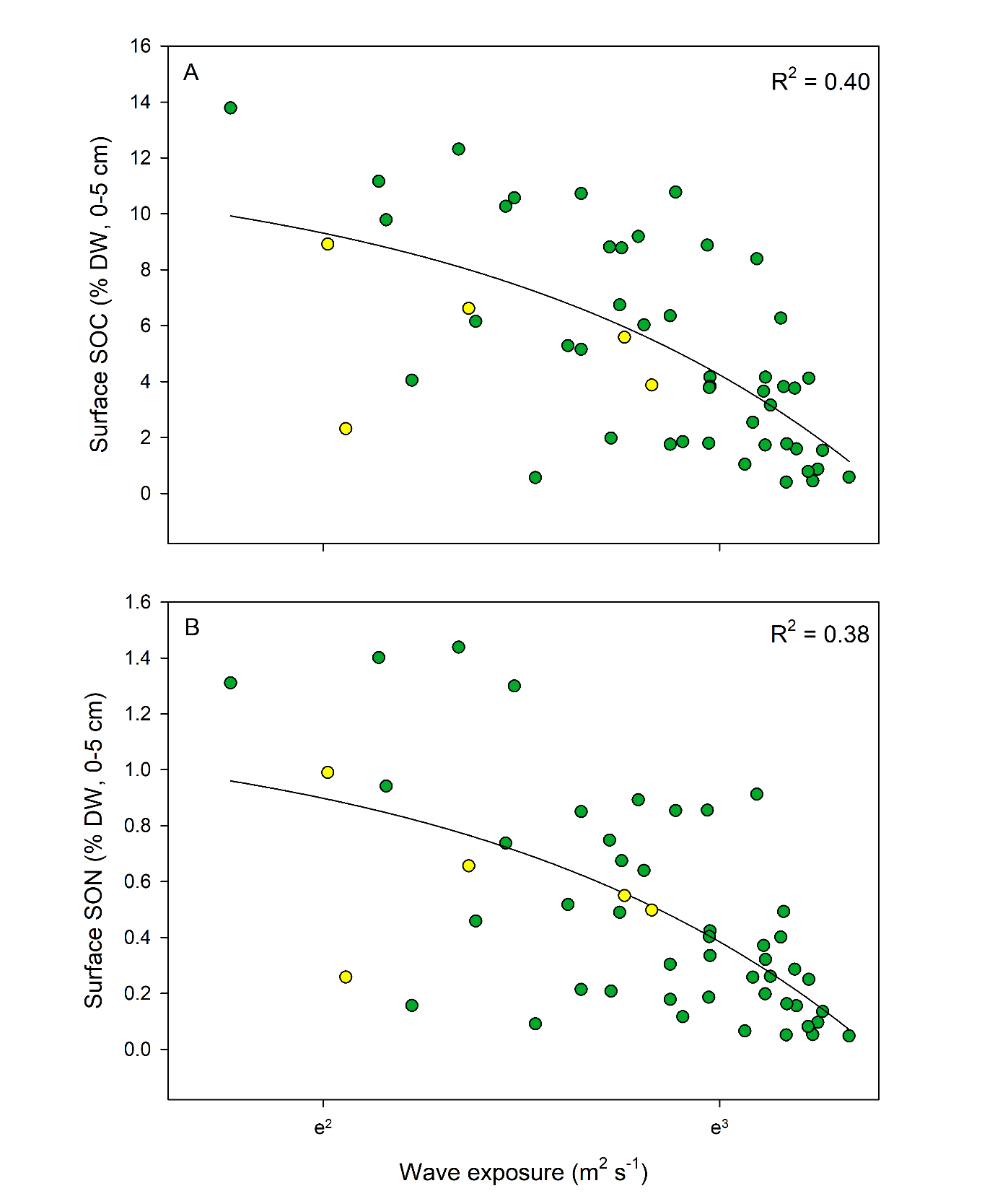
**

**Fig. S1.** Relationships between wave exposure and percent dry weight (DW) of surface sedimentary organic carbon (SOC) (A) and nitrogen (SON) (B) (0–5 cm) for all sites, except the two sites with lowest exposure values (n =51). The yellow dots (n = 7) represent the sites (the deeper part of the meadow) where the longer cores (0–60 cm) were collected.

**
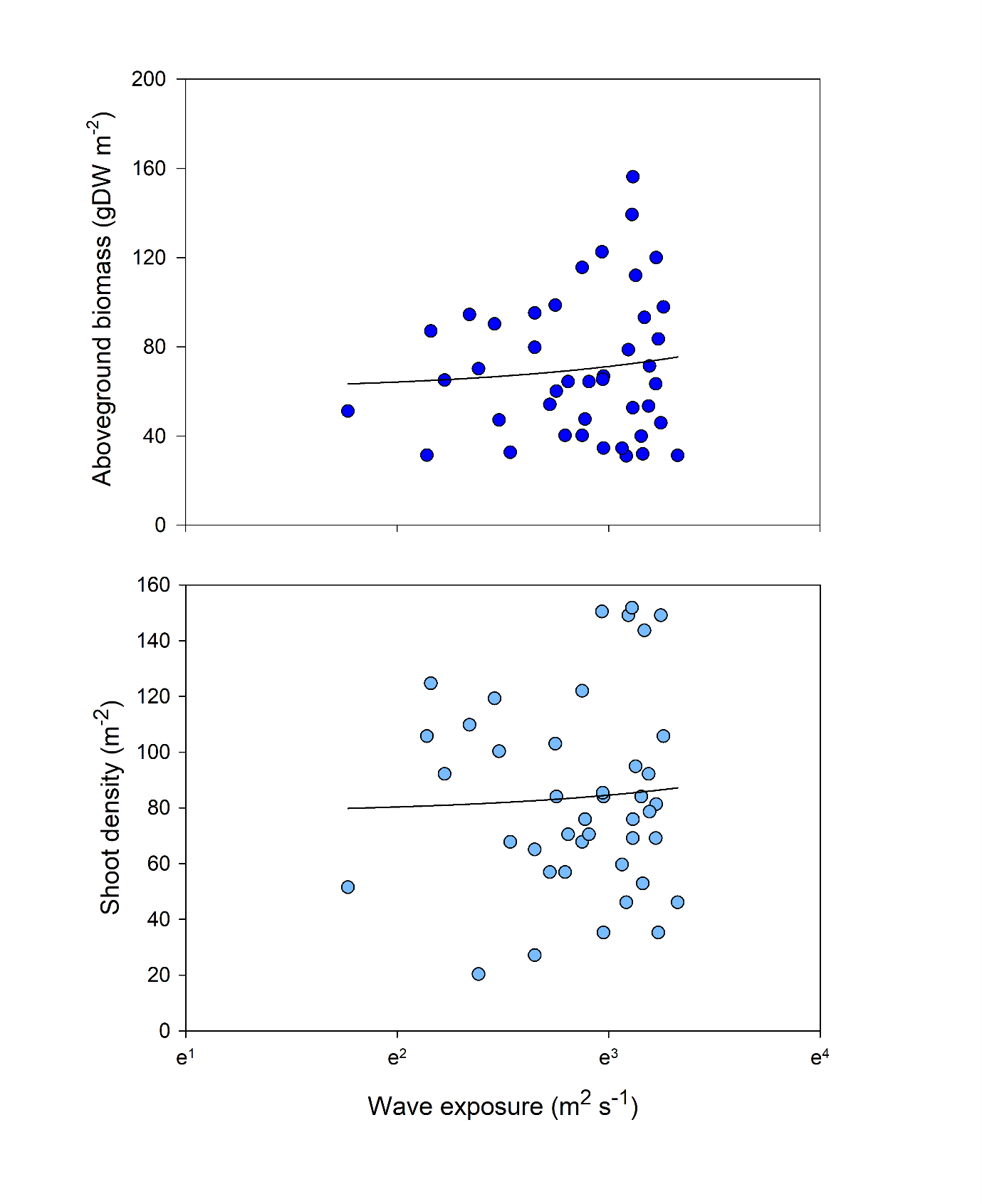
Fig. S2.** Wave exposure in relation to (A) aboveground seagrass biomass and (B) shoot density for the sites collected in 2000.

**
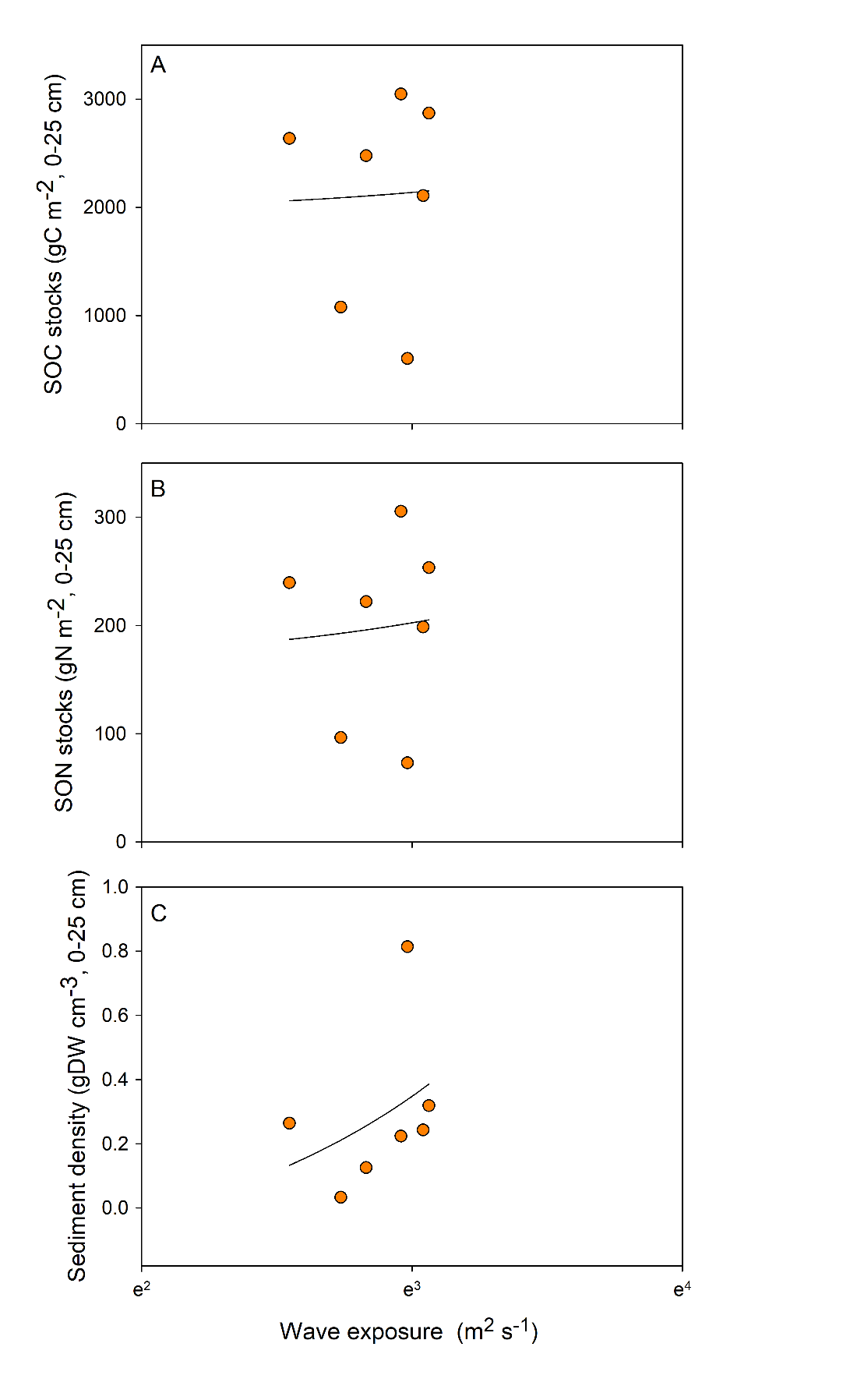
**

**Fig. S3**. Wave exposure in relationship to (A) SOC and (B) SON stocks (g m^-2^, 0-25 cm) for the shallow part of the meadow (n =7). The carbon and nitrogen stocks were corrected for sediment compression.


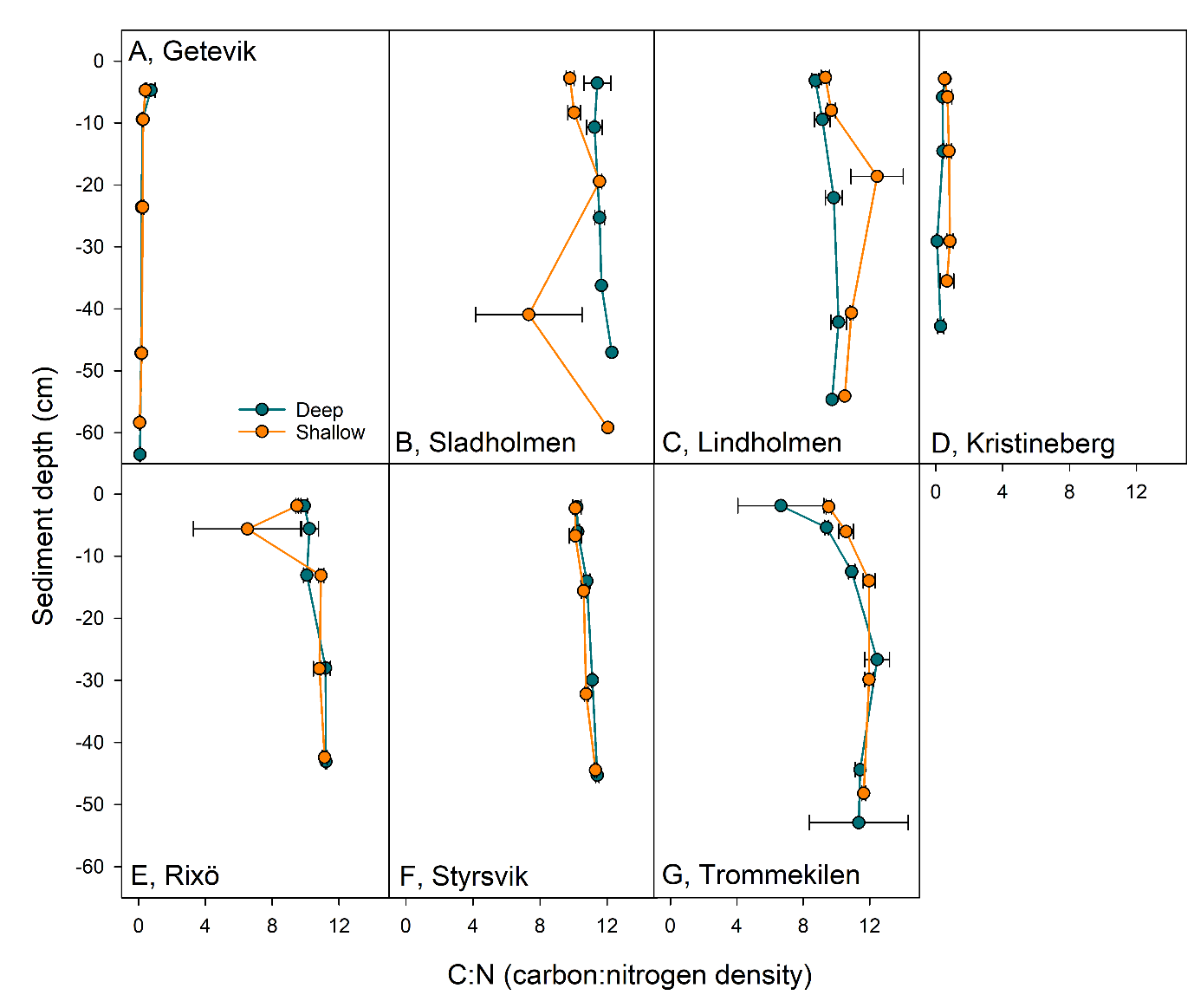


**Fig. S4.** Mean (±SE) sediment depth profiles of C:N-ratio (carbon:nitrogen density) in the deep and shallow parts of the meadows. The sediment length was corrected for sediment compression during sampling. SOC = sedimentary organic carbon.
